# Trait-dependent tolerance of bats to urbanization: a global meta-analysis

**DOI:** 10.1098/rspb.2018.1222

**Published:** 2018-08-22

**Authors:** Kirsten Jung, Caragh Grace Threlfall

**Affiliations:** 1 Institute of Evolutionary Ecology and Conservation Genomics, University of Ulm, Ulm, Germany; 2 School of Ecosystem and Forest Sciences, The University of Melbourne, Parkville, Australia; 3 School of Life and Environmental Sciences, The University of Sydney, Camperdown, Australia

**Keywords:** biodiversity, Chiroptera, conservation, functional traits, meta-analysis, urban ecology

## Abstract

Urbanization is a severe threat to global biodiversity, often leading to taxonomic and functional homogenization. However, current urban ecology research has focused mostly on urban birds and plants, limiting our ability to make generalizations about the drivers of urban biodiversity globally. To address this gap, we conducted a global meta-analysis of 87 studies, including 180 bat species (Chiroptera) from urban areas in Asia, Australia, Europe, North and South America. We aimed to (i) understand the importance of functional traits and phylogeny in driving changes in urban bat assemblages, and (ii) assess the capacity of traits for predicting which types of species are most sensitive to urbanization. Our results indicate that species-specific functional traits explain differences in the intensity of urban habitat use. Urban tolerance mainly occurred within the open and edge space foraging and trawling species as well as in bats with flexible roosting strategies. In addition, across bioregions and independent of phylogeny, urban tolerance correlated with higher aspect ratio, a trait enabling fast flight but less agile manoeuvres during aerial food acquisition. Predictive success varied between bioregions, between 43 and 83%. Our analysis demonstrates that the local extinction of bat species in urban areas is non-random, trait-based and predictable, allowing urban landscape managers to tailor local conservation actions to particular types of species.

## 1. Introduction

Urbanization is a leading cause of biodiversity decline globally [1], yet our understanding of the processes structuring urban biotic assemblages is limited. The question of which factors influence species tolerance or aversion [2], and thus the composition of urban assemblages, is central to urban ecology and conservation biology. Such understanding is necessary to facilitate the prediction of species response to urban-induced change, expand our knowledge of critical processes impacting upon urban biota and allow for actions to be identified that may assist effective urban conservation.

Several studies describe changes in the composition and structure of species assemblages associated with urban land-use [3–5], where this has predominantly been investigated for birds, insects and plants [3–7]. A global analysis of urban bird assemblages demonstrates that the density of bird species declines with increasing urban land cover [5]; however, many studies report increased density or richness of species with increasing urbanization, especially for plants [4]. These and other studies suggest that there may be traits common among species that benefit or decline in urban areas [8]. Species life history and functional traits are a useful tool to investigate general patterns in the response to environmental gradients across species [9], which may be especially useful in cities, as they share many environmental features such as high proportions of impervious surfaces, increased temperatures and reduced or fragmented natural habitats.

Studies examining plant traits suggest that few single traits show consistent responses to increased urbanization [8]. Instead, suites of traits may better explain urban tolerance across the wide variety of plant species found in cities [9–10]. A similar conclusion has been made in relation to studies of urban birds, where a combination of traits including habitat breadth, geographical range size, nesting location and number of breeding attempts have been shown to be important across species classified as ‘urban adapters or exploiters' [2,11]. These patterns may occur because the nature of the urban environment may enable certain species to have a high level of pre-adaptedness to urban living [12], allowing them to thrive across many cities (e.g. bird species within the families Sturnidae, Corvidae and Columbidae, which have successful urban representatives in most cities in which they occur [2]). Further, irrespective of the phylogenetic relatedness of species, specific functional traits may allow species to adaptively respond to urban environmental changes (e.g. via behavioural plasticity [12]). However, recent studies suggest that behavioural plasticity is less associated with urban tolerance than first thought [2,13], and instead traits associated with habitat breadth and generalist resource use are characteristic of urban tolerating species [2]. Importantly, these studies reiterate the need to conduct global comparative studies to be able to elucidate general ecological mechanisms responsible for structuring urban biotic assemblages [14], not only to further our understanding of the processes that influence species responses to urban impacts, but also to help identify the types of species for which targeted management and conservation may be most effective.

Bats (Chiroptera) are a diverse group of mammals that commonly occur in urban areas [15,16]. The high phylogenetic diversity of bats [17], in addition to the increasing number of studies examining urban bat communities [16], provides an opportunity to assess global patterns in trait filtering. These regional studies suggest that certain bat species seem to be better able to adjust to urban landscapes than others [16,18,19]. Although bat families differ in evolutionary history and geographical distribution [17], in certain regions urban-tolerating species have been found to have faster flight [18], greater mobility [20] and lower echolocation call frequencies [21]; however, the generality of these patterns across the globe is unknown. Further, in some areas, species with these traits reveal opposing responses to urbanization [22], suggesting further research examining trait-based responses across different regions is needed. Further, bats have been suggested to be suitable ecological indicators of urban impacts [15], and hence a global evaluation of the trait-based response of this group could help to promote targeted conservation, and shed light on the types of traits promoting or hindering different species.

The aim of this study is to assess the response of insectivorous bats to urbanization and assess the importance of functional traits in explaining species tolerance or vulnerability to urbanization across the globe. We used a meta-analytic approach, with a specific focus on aerial insectivorous bats that face the similar sensory challenge to navigate, detect and capture moving insect prey using echolocation.

We compiled published data of 180 species of insectivorous bats in urban and non-urban environments across five of the world's continents. We then asked (i) whether and how selected morphological and sensory traits correlate with urban tolerance in bats, (ii) if those traits are the same across species, and (iii) if traits can be used to predict bat species vulnerability to urban habitat change.

## 2. Methods

### (a) Data sources

We compiled peer-reviewed literature that investigated the effect of urbanization on abundance and activity patterns of insectivorous bats. A literature search was conducted in the Web of Science (Thomson Reuter) using the key words ‘bats’ AND ‘urban’, ‘urbanization’, AND ‘habitat’, ‘gradient’, AND ‘community’, ‘assemblage’, ‘species composition’, ‘presence’, ‘abundance’, ‘activity’ in different combinations, found in title, key words and abstracts. The literature search included publications up to May 2016. We also searched the reference lists of these publications for further relevant articles. This resulted in a total of 145 potentially relevant studies.

Meta-analysis is a useful tool that can provide new insights into questions that cannot be answered within a single publication. However, heterogeneity between original datasets is a challenge to any meta-analytic approach as it greatly influences the conclusion drawn and requires a careful selection of the primary studies. We were interested in the effect of urbanization on individual bat species and whether and how differences in urban habitat use between species correlate with functional traits. For our study, we thus specifically required publications that included data of individual bat species activity, abundance or occurrence in urban areas and thus excluded studies that focused on different biological questions (e.g. parasitation rate, roost selection, reproductive success or competition in urban areas), and review articles presenting non-original data. We further evaluated the definition of ‘urban’ within each publication and excluded papers that focused exclusively on habitat types such as national parks, forest fragments, parklands, waterways and roads within an urban landscape matrix to avoid interacting effects of additional environmental conditions which probably mask urban effects on bat species occurrences. In the remaining studies, we followed the original author definition of what represents an ‘urban’ area within the respective study region. In this way, the definition of ‘urban’ in our dataset varies between publications (see electronic supplementary material, Appendix table S1) from built-up city centres to residential areas, smaller settlements and urban backyards within residential areas.

To further evaluate the effect of urbanization on individual species occurrence and abundance, we required species-specific reports and thus omitted studies that did not present data of individual species and rather focused on species richness or diversity of bats in general. In some original publications, species-specific data were presented in graphs. If data could not be extracted directly from graphs (as is the case in regression trees or regression plots), we contacted the authors to contribute original data for our analysis. However, this could not be obtained in some cases (*N* = 4).

The type of data reported in the remaining 87 studies differed substantially; hence, we further split them into two different datasets. The first dataset comprised articles with quantitative and standardized data on flight activity, abundance or habitat use of aerial insectivore bats in urban versus non-urban habitat. This dataset allowed us to calculate a standardized effect size per species (*N* = 116) within each publication (*N* = 34), contrasting urban to non-urban habitats, respectively, and thus permitted the calculation of a standardized effect size across studies (see below) to conduct phylogenetic meta-regression models. If studies within this dataset reported the response of species to more than one habitat type in urban or non-urban landscapes, we extracted data from the highest degree of urbanization (e.g. city centres) and non-urban habitat (details are provided in the electronic supplementary material, Appendix table S1).

The majority of the studies included in the first dataset used acoustic monitoring and reported occurrence counts or flight activity of species during standardized sampling time between urban and non-urban areas. We also included a few studies (*N* = 6) that reported standardized and species-specific data on capture success in urban versus non-urban areas. Here, we assumed that reports of occurrence, flight activity or abundance/capture success measures all represent an indicator of habitat use and, if these measures were higher in urban versus non-urban areas, we assumed the species in question may ‘tolerate’ urban habitats.

The second dataset included various types of aerial insectivore bat species reports from urban areas only (*N* = 53 publications), including checklists, occasional sightings and reports from telemetry studies that presented raw foraging time data. Owing to the high variability in the type of data reported and the missing non-urban control within the respective study areas, we transformed all of these values into presence-only data. This dataset was exclusively used to test predictions from phylogenetic meta-regression models based on the first dataset. Here, we considered that if a species was present in an urban area, it could be classified as ‘tolerating’ urban habitats.

Both datasets together yielded in a total of 610 reports of 180 aerial insectivorous bat species, including the acoustic species complex *Eumops bonariensis/glaucinus* and the genus *Nyctophilus*.

### (b) Species classifications, traits and phylogenetic information

We classified species into taxonomic families and genera and assigned species according to their predominant foraging space (open, edge and narrow space) and foraging mode (aerial hawking, gleaning, flutter detection, trawling) into five functional guilds following [23]. We further classified species into roosting generalists or specialists. Hereby, we only considered natural roosts (caves, rocks and crevices, tree hollows, bark and trunk, foliage and plant structures) based on the most common term in the literature (following [24]) and excluded reports from artificial roosts such as bat boxes or roosts in buildings as these represent human influence, which is always greater in urban compared with non-urban areas. If a species was only reported roosting in one of such categories listed above, we considered it as a roosting specialist; if it was reported to use various types of roosts, we classified it as a roosting generalist. From the IUCN Red List of Threatened Species (IUCN 2017), we obtained the conservation status and checked individual species names for potential synonyms.

Classifications, however, only allowed us to derive observational patterns of predefined categorical classes. We thus further gathered continuous morphological and sensory trait information (see electronic supplementary material, Appendix table S2), including forearm length, weight, aspect ratio (the ratio of wing span to wing area), wing loading (wing area per body mass), peak frequency (frequency of maximum amplitude) or characteristic frequency (in the case of zero-cross-based recordings, i.e. Anabat recoding systems) and duration of echolocation calls. Besides forearm and weight as two complementary measures for body sizes of bat species, these traits are considered to directly affect mobility, agility and prey perception in aerial hawking bats. While wing loading and aspect ratio positively correlate with flight speed and thus suggest higher mobility during nightly foraging events [25], echolocation call frequency and call duration determine sensory constraints during foraging, such as the perceptual range of echolocation due to atmospheric attenuation and the detection of prey items in different acoustic clutter situations [26]. All of these traits are frequently assessed during field surveys (standard mist-netting and acoustic surveys) and could be obtained from museum specimens and acoustic sound libraries. The trait dataset presented here is the first compilation of urban bat traits that incorporates multiple aspects of bat biology, including resource requirements and morphological and sensory environmental constraints.

To evaluate phylogenetic relatedness between bat species within taxonomic units, we employed the species-level supertree of bats [27], which is based on the previous published tree [28,29]. For each species that was missing from the tree (*N* = 3 of the 116 species), we used the closest congener present. The supertree approach takes topologies created from primary character data and combines them to form a more comprehensive phylogeny [30]. Although it may lack details of individual systematic studies, which are currently still limited to subgroups of bats or certain regions, it allows a macro-scale comparative analysis to understand evolutionary patterns and processes of trait data [27].

### (c) Data analysis

#### (i) Calculation of effect size

Based on the first dataset, we compared the relative intensity of habitat use per species within each publication in urban (treatment group) versus non-urban (control group) areas and calculated the log odds ratio as a standardized effect size, which allows for between-study comparison [31]. To account for additional heterogeneity in species response to urbanization between studies, we further conducted a ‘random effect model meta-analysis' which considers that our data represent a random subset of potentially more, not yet available, data [32]. This analysis gave us an estimated effect size and standard error per species, which we used for meta-regression analysis. A positive estimate (effect size: greater than 0) indicated a higher intensity of habitat use in urban areas (as indicated by greater occurrence, flight activity, abundance or capture success) and thus likely a higher tolerance to urban areas, compared with a negative estimate (effect size: less than 0), which indicated a higher intensity of habitat use in non-urban areas.

#### (ii) Meta-regression and prediction of urban tolerance

We then fitted random effects meta-analytic models (function: rma.mv, package metaphor [32]) to investigate the overall effect of urbanization. Overall results therein are presented as a test of heterogeneity (*Q*), where a significance level of *p* < 0.05 indicates that the heterogeneity in the response significantly deviates from zero and thus habitat use significantly differs between urban and non-urban areas. To deal with a possible dependence of species effect sizes due to phylogenetic relatedness [33] and biogeographic regions, we conducted a second model including the bat phylogeny and continents as a random effect. This three-level meta-analytic model allowed us to test how much heterogeneity in the response of bats to urbanization can be attributed to species-specific differences, phylogenetic relationships between species and biogeographics regions [34,35]. Relative importance of random effects are given by ‘*σ*’. To assess whether it was necessary to account for phylogeny and biogeography, we then used one-sided log-likelihood-ratio tests to compare the fit of the original three-level model with the fit of a reduced model in which the variance of either phylogeny or biogeography was set to zero.

We further assessed whether species traits moderate the overall response to urbanization (test of moderators, results presented as ‘QM’) and whether species tolerating urban areas worldwide (independent of their phylogenetic relatedness and biogeographics occurrence) share similar traits. We thus fitted traits as fixed factors into the model. In addition, we included the phylogeny nested within bioregions (continents) as a random factor, as pre-analysis (function Adonis, package vegan) revealed that closely related species shared similar functional trait characteristics (table 1). Here, we analysed each categorical trait (functional guilds, IUCN status, roost) separately, because in meta-analysis, multi-factorial analysis does not allow for an assessment within factor levels (e.g. within each category).

**Table 1.** Correlation of categorical and continuous traits with phylogenetic relatedness of bat species. Closely related bat species share similar functional traits.

traits	*F*_4,73_	*R*^2^	*p*-value
functional guilds	9.12	0.27	<0.001***
IUCN status	0.93	0.01	>0.05^n.s.^
roosting specialization	2.70	0.02	<0.05*
forearm (mm)	8.16	0.06	<0.01**
weight (g)	8.33	0.06	<0.001***
wing loading	3.03	0.02	<0.05*
aspect ratio (%)	6.01	0.04	<0.01**
echolocation frequency (kHz)	6.90	0.05	<0.001***
call duration (mm)	0.88	0.01	>0.05^n.s.^

We further tested which of the continuous morphological and sensory traits were adequate predictors for species tolerance in urban areas using the function ‘predict’. This function provides predicted average effect size and 95% confidence intervals for stepwise increasing absolute trait values while providing the option of holding another trait constant. This allowed us to correct for size differences between bat species, which is important for bats, because wing morphological measurements such as aspect ratio and wing loading, and echolocation parameters, are not independent of body size [25,36]. Hence, we used forearm length as an indicator of body size, as it is a less variable parameter between captured individuals compared with body mass, can be obtained by museum specimens and is frequently used for species identification in bats.

Predictions were then conducted for a median-sized bat across all bat species in our first dataset with a forearm length (40 mm) and the first and third quartiles (35 mm/47 mm), while testing the effect of applying a stepwise increase in the other above-listed trait parameter values. This allowed a more accurate prediction of trait characteristics for urban-tolerant species. To test for potential differences in the predictive power of continuous traits between bioregions, we repeated the above predictions within each of the five continents.

In cases where species trait data were missing (see electronic supplementary material, Appendix table S2), we excluded the species from that particular regression and predictive analysis.

Finally, we extracted the predicted trait parameter values that may distinguish bats likely to forage in urban areas from urban-avoiding species for a median-sized (40 mm), a smaller (35 mm) and a larger bat (47 mm). All critical trait values were extracted at a conservative probability threshold of 75% and a less conservative threshold of 60% likelihood to tolerate urban areas, indicating that a species with a respective trait parameter value is 75% (or 60%) more likely to occur in urban areas, compared with species not having this trait value. If predicted trait values did not coincide with the natural range of the respective trait in our dataset, we considered those predictions as non-applicable.

#### (iii) Testing predictions using the second dataset

Our second dataset included only species presence data in urban areas and their species-specific traits (see electronic supplementary material, Appendix table S3). Using this dataset, we then derived the proportion of bat species characterized by traits that we predicted to enable tolerance to urban areas. Here, we first evaluated each trait separately and then assessed the predictive success of combining the results of categorical traits with predicted parameter values based on continuous functional traits.

For all statistical analysis, we used the statistical software package R v. 3.3.1. [37]. Meta-analysis was conducted using the package metaphor [38] (v. 2.0-0).

## 3. Results

### (a) Effect of urbanization on bats

**Figure 1.**
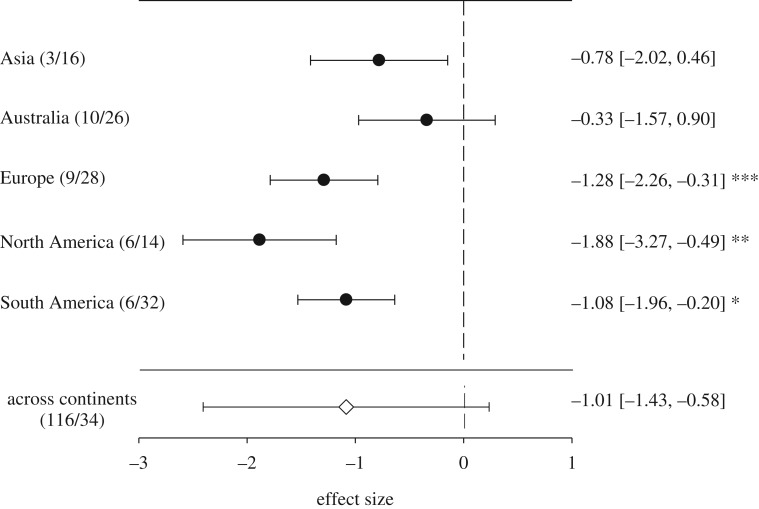
Differences in the overall response of bat species to urbanization (accounting for phylogenetic non-independence of species responses) and separated by different bioregions. The number of publications and species per continental assemblage is given in parentheses. Plotted are the estimated effect sizes and standard errors per continent. Values of the estimated effect size, including the 95% confidence intervals, are listed on the right side of the figure. *p*-values: ‘***’ = 0.001, ‘**’ = 0.01, ‘*’ = 0.05.”

Random effect model meta-analysis revealed an overall significantly negative effect (estimate: −1.07, s.e.: 0.25) of urbanization on aerial insectivorous bats (deviance = 551.18, *z*-value = −4.31, *p* < 0.0001). These overall results indicated a 34% (CI 21–56%) reduction in the intensity of habitat use in urban areas compared with non-urban areas. However, the strength of this effect varied extremely between individual species (*Q*_(d.f.__=__115)_ = 434.01, *p* < 0.0001), indicating high variability in the ability to tolerate urban areas (see electronic supplementary material, Appendix figure S1). In addition, standard errors of effect sizes indicated a certain flexibility in habitat use for several species. Only some particular species appeared to either significantly avoid or tolerate urban habitat. Including phylogeny and bioregions as random effects into the model supported the negative effect (estimate −1.10, s.e.: 1.86) of urbanization but indicated a higher variance in species response (deviance = 543.1, *z*-value = 0.59, *p* > 0.05). Phylogeny (*σ*^2^ = 0.12) and bioregions (*σ*^2^ = 0.14) together explained about 17% of the total heterogeneity in species response. While log-likelihood-ratio tests confirmed a significant contribution of phylogeny (LRT 173.45; *p* < 0.001) as a random factor to the overall variance in species response to urbanization, including bioregion as random factor increased the variance (figure 1), but did not change the overall significantly negative effect (estimate: −0.81, s.e.: 0.29) of urbanization worldwide (LRT = 0.38, *p* > 0.05). Across bioregions, however, the effect of urbanization differed, where it appeared less pronounced in Australia when compared with other regions (figure 1).

### (b) Moderating effects of functional traits

Meta-regression revealed that, independent of phylogenetic relatedness and biogeographics occurrence, species potential to tolerate urban areas correlated with categorical (QM_(d.f.=4)_ = 13.0696, *p* = 0.01) and continuous functional traits (QM_(d.f.=6)_ = 32.1503, *p* < 0.0001).

**Figure 2.**
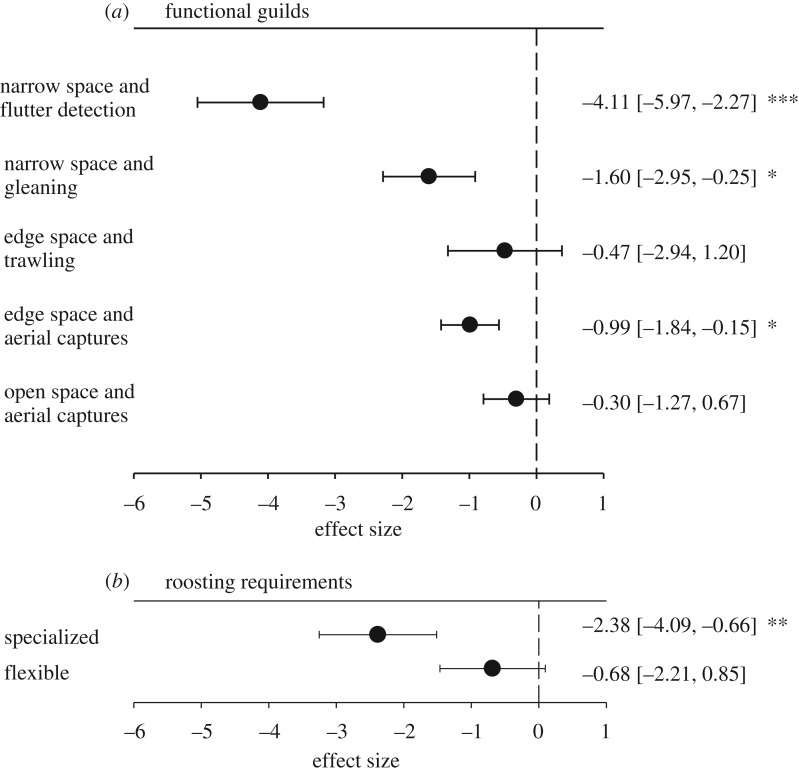
The effect of urbanization (estimated effect size and standard error) on bat species classified into different functional guilds and according to roosting requirements. A negative effect size reflects a higher association with non-urban areas; a positive effect size reflects an association with urban areas. Values of the estimated effect size, including the 95% confidence intervals, are listed on the right side of the figure. *p*-values: ‘***’ = 0.001, ‘**’ = 0.01, ‘*’ = 0.05.”

In particular within the classification of narrow space flutter detecting (estimate: −4.12, s.e.: 0.95, *p* < 0.0001) and gleaning species (estimate: −1.6, s.e.: 0.69, *p* < 0.01), as well as edge foraging aerial insectivores (estimate: −0.99, s.e.: 0.43, *p* < 0.05), urban tolerance was significantly less likely compared with open space aerial foraging and trawling species (figure 2*a*). In addition, species specialized to certain roosting types (estimate: −2.38, s.e.: 0.8761, *p* < 0.001, figure 2*b*) occurred significantly more often in non-urban areas, compared with species with flexible roosting requirements. Urban tolerance was not associated with IUCN status.

**Figure 3.**
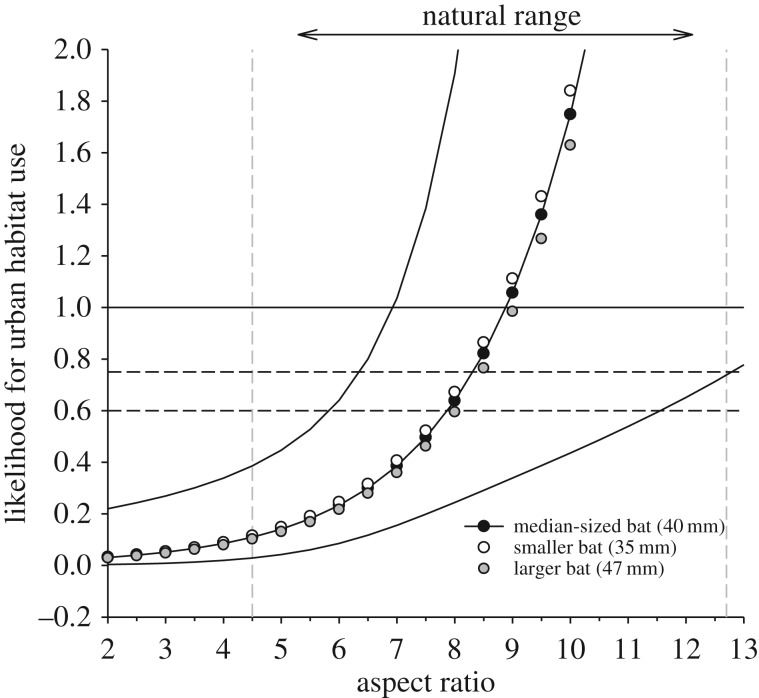
The predicted likelihood of urban habitat use based on aspect ratio. Predictions were conducted for a median-sized bat across all bat species in our first dataset with a forearm length (40 mm) and the first and third quartiles (35 mm/47 mm). Confidence intervals are indicated by the two black lines. Likelihood values above 1 suggest urban tolerance; values below 1 indicate lower tolerance towards urban areas. Likelihood thresholds of 60% and 75% are indicated by the horizontal grey dashed line and suggest that a species with a respective trait parameter value is 60%/75% more likely to occur in urban areas, than a species not having this trait parameter value. The natural range of aspect ratio in our first dataset is indicated by the two vertical dashed lines.

In the majority of cases worldwide, urban-tolerating species were characterized by shorter forearm length (*p* < 0.01), higher aspect ratio (*p* < 0.05) and higher weight (*p* < 0.05). Hereby, our results also indicated a significant interaction (*p* < 0.01) between forearm length and weight, pointing to a non-independence of both parameters and suggesting that heavier bats at a given size (and thus faster flying species) predominantly occur in urban areas. Predicted effect size further indicated that, independent of their phylogenetic relatedness or biogeographics distribution, a higher aspect ratio (QM_(d.f.=1)_ = 7.77, *p* < 0.01) at a given body size remained a significant and meaningful predictor of urban tolerance across species worldwide (figure 3).

Predictions within each bioregion additionally suggested a tendency of increased tolerance to urban areas for species characterized by higher weight (South America, Australia), higher wing loading (North America, Australia), lower echolocation call frequencies (Asia, Australia, North America) and longer call duration (Australia, South America). In addition within North America, significantly smaller species (QM_(d.f.=1)_ = 6.9, *p* < 0.01) occurred in urban areas, while in Europe our analysis indicated only a significant correlation of urban tolerance with increasing aspect ratio (QM_(d.f.=1)_ = 4.3, *p* < 0.05).

### (c) Testing predictions on an independent dataset

Predicted tolerance to urban areas based on aspect ratio at a median forearm reached a predictive success of 41–68% (except for Africa), depending on the respective bioregion (table 2). Limiting this analysis to open and edge aerial foragers and trawling species, and incorporating roosting requirements, further increased the predictive success to 42–83% in our test dataset.

**Table 2.** Extrapolated trait parameter values for urban tolerance and classification success per continent, based on predicted effect size of aspect ratio for a larger (greater than 40 mm forearm) and/for smaller (less than 35 mm forearm) bat species. Predicted aspect ratio was extracted at a 75% and 60% likelihood to tolerate urban areas (see also figure 3), suggesting that a species with this predicted aspect ratio value is 75% or 60% more likely to occur in urban areas compared with a species with lower aspect ratio values. Classification success of species in the second dataset based on the derived predictors is given on the right side of the table per continent (*N* species) and for different predictor combinations: (i) aspect ratio as the only predictor, (ii) aspect ratio, guild classification and roosting specialization combined.

	predicted trait parameter value		classification success %					
predictors	threshold (%)	larger/smaller bat	Africa (17)	Asia (17)	Australia (18)	Europe (14)	N-America (12)	S-America (19)
aspect ratio	75	8.3/6.9	5.8	35.3	55.5	35.7	8.3	57.9
	60	7.9/5.8	23.5	52.9	61.1	57.1	41.6	68.4
traits combined	75	8.3/6.9	9.1	50.0	66.6	50.0	14.0	83.0
	60	7.9/5.8	27.2	70.0	73.3	70.0	42.8	83.0

## 4. Discussion

Maintenance of biodiversity in urban areas requires an in-depth understanding of the deterministic processes which are responsible for species tolerance to increasing urbanization [2]. Using a comprehensive global dataset on insectivorous bat species, combined with an extensive compilation of morphological, sensory and behavioural bat traits and phylogenetic information, our results support the hypothesis that urbanization leads to a trait-based environmental filtering of insectivorous bat species at a global scale. In addition, we show for the first time that certain traits successfully predict the potential of species tolerance to urban habitat change.

Across continents, our study revealed that urban tolerance in bats correlates with traits related to greater flexibility in resource requirements and greater mobility. In particular, we found species that tolerated urban areas were generally open and edge aerial foragers and trawling bats, with flexible roosting strategies and species with higher weight and aspect ratio (narrow-shaped wings) at a given body size. These species might take advantage of increased roosting opportunities such as bat boxes or buildings in urban environments. In addition, the higher weight and aspect ratio in relation to other bats with similar body size, likely correlates with higher flight speed or the ability to sustain longer flights. These flight characteristics may allow these species to maintain or even increase foraging efficiency, although resources are likely scattered across a city, concentrated at street lights [39], urban parks [18,40] or over water [41,42] and thus likely require longer commutes or longer foraging activity per night.

Within bioregions, our analysis further suggested an increased tolerance to urban areas for species characterized by higher weight, higher wing loading, lower echolocation call frequencies and longer call duration. However, the importance of these traits differed between bioregions. Individually, these traits have been previously named as correlates with urban tolerance in several regions [16]. However, extant bat species differ in evolutionary history and biogeographics distribution, thus results of regional studies cannot be extrapolated to evaluate the consistency of trait correlations globally. Additionally, global comparisons are needed to evaluate the importance of phylogeny for urban tolerance. This study is the first to ask if urban tolerance of bats can be predicted at a global scale, using a method that allowed for the inclusion of a broad suite of traits, while accounting for differences between bioregions and the influence of phylogeny. Using this approach, we show that mainly three globally consistent traits correlate with urban tolerance of bats across bioregions and that these successfully predict the likelihood of a species to tolerate urban areas, greatly improving our ability mitigate the negative impacts of urbanization and set conservation priorities.

Our findings agree with published results for birds, butterflies and plants [2,43], where traits related to mobility, resource requirements and specialization explained the difference between urban tolerance and urban avoidance. Being highly mobile, and able to disperse to find areas of favourable resources is commonly thought to lead to lower sensitivity to habitat loss [44]. For example, plants with heavier seeds [10], and those able to be transported by water or via ingestion by animals, have been associated with urban tolerance or colonization [10,43].

Concepción *et al*. [43], however, showed, that even highly mobile species can be negatively affected by urban habitat change if they depend on specific resources, that may be rare or unavailable in the urban landscape. In particular, highly mobile but specialized birds and butterfly species that relied on natural vegetation, and those unable to exploit resources provided by urban vegetation have been found to decline with increasing urbanization [43]. Similarly, Sol *et al.* [2] demonstrate that birds with narrower habitat requirements (e.g. more specialized species) were most likely to decline in urban areas, and Palma *et al.* [10] show that plants with unspecialized nutrient requirements characterized urban colonizing species. While we found this was the case in relation to roosting strategies for bats, where those with specialized requirements were negatively associated with urban areas, specialization of aerial insectivore bats to certain prey types is largely unknown and thus could not be included in our analysis. Nevertheless, it might explain why, for example, many of the highly mobile *Eumops* species in the Neotropics, known to predominantly roost in trees and specialize on beetles [45], revealed a negative response to urban areas.

Our results revealed an overall negative effect of urbanization globally. However, the strength of this effect varied between bioregions and individual species, where some species actually increased their use of urban versus non-urban habitats. Five species were found to significantly tolerate urban habitat: *Vespertilio murinus* from Europe, *Scotophilus kuhlii*, *Scotophilus heathii* and *Taphozous melanopogon* from Asia and *Molossus molossus* from South America. These species all have relatively high aspect ratios, flexible roosting strategies and are open or edge space foragers, suggesting that these species may exploit urban habitats and could potentially expand their range, as has been found for other bat species [46,47].

For the most part, our analysis indicated these patterns in tolerance or avoidance of urban areas were species-specific, and only in some cases do related bat species (e.g. genera, families) respond in a similar way. For example, in certain regions, we found strongly positive or negative responses in the genera *Noctilio* (estimate: 1.1, s.e.: 0.9) and *Molossus* (estimate: 1.0, s.e.: 0.7) in South America, *Rhinolophus* in Asia, Australia and Europe (estimates: −3.1, s.e.: 0.6) and *Myotis* in Europe (estimate: −1.3, s.e. 0.5). For most species included in this analysis, however, response to urban habitats was not associated with phylogeny. Nevertheless, many of the traits tested here are significantly nested within the bat phylogeny, indicating an indirect effect of evolutionary history on the likelihood of species to tolerate urban areas via functional traits [48]. Thus, in agreement with the ‘urban tolerance hypothesis' [2], species with traits that allow for foraging in open space are more pre-adapted for a life in the city than others. Conversely, species that do not possess these traits are probably at risk of decline in urban areas, and hence should be the subject of local targeted conservation actions. Indeed, bat species likely to decline are those that rely on vegetation for roosting (tree hollows and plant structures) and foraging opportunities (gleaning and flutter detecting species), and the retention of large patches of original vegetation in cities would not only benefit these bat species, but also many species of plants, birds and invertebrates [49]. Thus, the methods used here to identify the types of species most vulnerable to increasing urbanization may be usefully applied to other taxa to identify whether commonalities between taxa exist, and elucidate whether common conservation measures can be applied at large scales.

It must be noted that our analysis suggested a high variability in the predictive success between bioregions. This may reflect the unequal distribution of studies in different bioregions (e.g. missing publications contrasting urban versus non-urban areas from Africa), the differing level of urbanization between bioregions, or that our dataset missed an important additional trait that may help to explain urban tolerance, such as dietary specialization. Further, both datasets we used contained data obtained using different sampling methods (acoustic surveys, capture and telemetry data). While we carefully selected publications presenting standardized sampling between urban and non-urban areas for the first dataset and calculated log odds ratios for meta-regression analysis per species within each publication to account for differences in species detectability due to different sampling methods, our test dataset consisted of a highly heterogeneous data (including checklists of species occurrences), which we transferred into a presence-only data (as discussed by Sol *et al*. [2]). Hence, our test dataset did not discriminate between species frequently observed in urban areas and those that are rare. As such, our results of predictive success are probably conservative, and future monitoring of urban bat populations using standardized approaches between urban and non-urban areas should be employed to assess species persistence over time, especially in data-deficient bioregions such as Africa and Asia.

Finally, the specific components of the urban environment that produced the presented patterns here are many and varied; thus, our results may include responses to artificial night light, increases in the density of predatory or competitive species and varied preferences for man-made habitat, among other factors. For example, increased night light is known to increase insect aggregations, which is often reported to benefit fast-flying bat species [50]. Further, the ability to switch roosts or exploit man-made structures for roosting may influence the extent and frequency of interspecific interactions that bats experience at the roost (e.g. with potential roost competitors [51]). Future research would benefit greatly from understanding the effect of these additional factors on urban bat assemblage composition. This avenue of research has indeed been recently suggested as a way of providing a second line of evidence to demonstrate environmental filtering [52], where studies should attempt to correlate changes in community structure, as demonstrated here, with specific environmental gradients found within urban areas, such as changes associated with varying intensities of urbanization and habitat availability. This type of analysis was outside the scope of this study, but may help to explain the differences between bioregions that we found, including the apparent differences in the magnitude of effect of urbanization between Europe and Australia. Future analysis of these patterns incorporating environmental gradients within cities is underway, and may clarify these patterns further, and assist in demonstrating where conservation efforts need to be targeted globally and at continental and regional scales.

## 5. Conclusion

To our knowledge, this is the first global test of the ‘urban tolerance’ hypothesis for bats, using a comprehensive dataset incorporating species traits and phylogenetic information. We found clear evidence that urban areas are filtering bat species based on traits, a pattern that is undetectable using locally restricted datasets of single cities, or continental analyses, which currently dominate the urban ecology literature [5]. In agreement with Sol *et al*. [2], we found that there are many bat species that do not possess traits that allow them to thrive in cities, and those that do generally have specific morphological, sensory and behavioural traits, leading to greater functional rather than phylogenetic similarity at the global scale. This increased functional similarity is of great ecological concern as a decrease in functional diversity also leads to a breakdown of species interactions necessary for ecosystem services [53]. Using traits to predict the vulnerability of bats and other taxa in cities, in addition to monitoring abundance, may thus allow practitioners to identify priority areas for conservation efforts within the urban landscape for both mobile and less mobile (non-flying) species.

## Data accessibility

The datasets supporting this article are available from the Dryad Digital Repository at: http://dx.doi.org/10.5061/dryad.84q03k0 [54].
